# Factors Affecting Unscheduled Return Visits to the Emergency Department among Minor Head Injury Patients

**DOI:** 10.1155/2017/8963102

**Published:** 2017-09-05

**Authors:** Kuo-Cheng Wang, Chung-Hsien Chaou, Peng-Huei Liu, Cheng-Yu Chien, Ching-Hsing Lee

**Affiliations:** ^1^Department of Emergency Medicine, Chang Gung Memorial Hospital, Linkou, Taiwan; ^2^Chang Gung University College of Medicine, Taoyuan, Taiwan; ^3^Chang Gung Medical Education Research Center, Taoyuan, Taiwan; ^4^Department of Emergency Medicine, Chang Gung Memorial Hospital, Taipei, Taiwan; ^5^Department of Emergency Medicine, Ton-Yen General Hospital, Hsinchu, Taiwan; ^6^Department of Emergency Medicine, Chang Gung Memorial Hospital, Keelung, Taiwan

## Abstract

**Study Objectives:**

Differences between returning and non-returning minor head injury (MHI) emergency department (ED) patients, between the characteristics of the first visit and revisit, and between admitted and nonadmitted returning patients were investigated.

**Methods:**

This was a retrospective study. All discharged ED patients with ICD-9 codes 850.0 to 850.9, 920, and 959.01 in 2013 were enrolled. Patients' demographic data, vital signs, Glasgow Coma Scale, ED diagnosis, length of stay, triage levels, ED examinations performed, and comorbidities were recorded for analysis.

**Results:**

A total of 2,815 patients were enrolled. Of 57 (2%) patients who revisited the ED, 47 (82%) were discharged from the ED and ten (18%) were admitted to the hospital. Patients who returned to the ED were older, and they exhibited more comorbidities. Those who presented with vomiting, triage level of 1 or 2, and GCS score of <15 and who received more blood tests during their first visit were more likely to be admitted when they returned to the ED.

**Conclusions:**

Discharging MHI patients who are older or exhibit comorbidities only when symptoms and concerns are relieved completely, providing clear discharge instructions, and arranging timely clinical follow-ups may help reduce such patients' return rate.

## 1. Introduction

Unscheduled return visits to the emergency department (ED) are regarded as an important indicator of treatment quality [1–3]. The underlying causes of such revisits may be premature discharge following the first ED visit, missed diagnoses, or treatment failure. With returning patients, medical resources are consumed to provide additional examinations, observations, or reassurances, which might further increase expense or cause ED overcrowding [4]. Some authors categorize the etiologies of return visits into patient-related factors, illness-related factors, system-related factors, and clinician-related factors [5]. The previously identified influential factors behind unscheduled ED return visits among the general patient population include older age, urgent triage level, presenting during the night shift, and certain types of initial chief complaints [6–9]. However, many studies have revealed that return patterns may be different among specific patient populations [10–12].

Minor head injury (MHI) is a frequent complaint in the ED. Most of these patients are discharged after initial management. Among those who exhibit no initial life-threatening problems and who are hence discharged, it is not uncommon for MHI patients to return to the ED, whether because of disease progression or worsening symptoms. In the previous literature, there have been very few studies that discussed the issue of unscheduled return visits to the ED on the part of MHI patients and often analyzed only a specific subgroup of minor trauma patients [13]. In this study, we aimed to investigate the factors that influence unscheduled return visits to the ED and return admissions among all MHI patients and to discuss the current principles behind managing this patient population.

## 2. Materials and Methods

### 2.1. Study Design

This study involved retrospective data analysis. The patients were enrolled from the ED of Linkou Chang Gung Memorial Hospital (LCGMH), a 3,600-bed tertiary teaching hospital with a Level I trauma center in Northern Taiwan that receives approximately 160,000 visits annually. The study was approved by the institutional review board at the focal institution and it qualified for a waiver of informed consent.

### 2.2. Study Population

Our study included all MHI patients discharged from the ED without surgery or admission who were issued the following ICD-9 codes: 959.01 (head injury), 920 (contusion of scalp), and 850.0~850.9 (concussion) in the study period (1 January 2013 to 31 December 2013). The returning patients were defined as those who returned to the ED within 72 hours following discharge. According to the protocol for minor head injuries, all of these patients received an instruction sheet and a follow-up appointment was arranged for each of them at a neurosurgical outpatient clinic within three to five days. Those who left against medical advice, who were scheduled to be transferred to another hospital, who left without being seen, or who left without notice were excluded.

### 2.3. Data Collection and Definitions

The electronic medical records of all the MHI patients enrolled were extracted from the hospital's database. The recorded data included age, gender, ED diagnosis, length of stay in the ED, 5-level triage level, initial body temperature, initial heart rate, initial mean arterial pressure, initial respiratory rate, initial Glasgow Coma Scale (GCS), whether blood testing was done, whether a brain computed tomography (CT) scan was performed, and whether an X-ray was taken. Brain CT scans were arranged according to the guidelines of the Canadian CT Head Rule [14] or the CT Rule for Minor Traumatic Brain Injury established by the Taiwan Neurosurgical Society. The length of stay in the ED was measured from the time of registration to the time of leaving. Information regarding previous comorbidities was also collected from the electronic medical database. We gathered data on administrative factors, including physician's gender, physician's seniority, total number of ED patients on the day of visit as an overcrowding variable, and whether the visit was during nonoffice hours. The night shift was defined as after 5 pm and before 8 am.

To identify the returning patients, we cross-matched the MHI patients with our 72-hour returning patient lists, which were routinely recorded as an ED quality control measure. Those who returned with a different chief complaint were excluded. If a patient came back more than twice, only the initial visit and the first return visit were included in the analysis. For the returning patients, we reviewed the medical records and collected the following data: (1) mechanism of trauma, categorized into traffic accident, fall, fight, and other; (2) initial symptoms, including loss of consciousness, vomiting, amnesia, and whether head and neck hematoma was present; (3) second visit conditions and management, including triage vital signs, GCS, triage level, and whether X-rays, blood tests, CT, and a consultation were conducted; (4) other factors, including whether the patient arrived by ambulance and whether there was documented alcohol use prior to the incident; (5) whether the patient received surgery; and (6) ED disposition, including discharge from the ED and admission to a ward or the ICU. The final outcome of admitted returned patients was classified into either discharge after admission or mortality.

The primary outcome is the difference between the returning and the non-returning patients. The secondary outcomes were the difference between the first and the second visit and between the admitted and the discharged returning patients.

### 2.4. Statistical Analysis

All data were expressed as mean ± standard deviation or number (percentage). Continuous variables from two independent groups were analyzed using the independent *t* test, while categorical variables from independent groups were analyzed using either the *χ*^2^ test or Fisher's exact test as appropriate. A paired *t*-test or a McNemar test was used for paired continuous or categorical data, respectively. All data were analyzed using SAS statistical software Version 9.2 (SAS Institute, Inc., Cary, NC). A value of *p* < 0.05 was considered to be statistically significant.

## 3. Results

### 3.1. Inclusion, Exclusion, and Patient Baseline Characteristics

**Figure 1:**
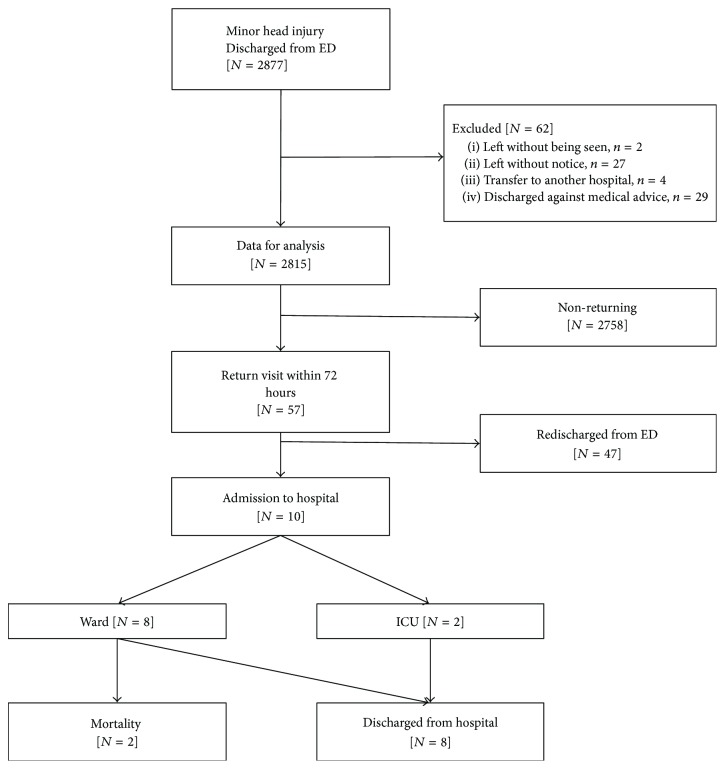
Flow chart for inclusion and exclusion.

A total of 2,877 MHI patients were discharged from the ED. Sixty-two patients were excluded from the study, including two who left the ED without being seen, 27 who left without notice, four who transferred to another hospital, and 29 who were discharged against medical advice. Out of the remaining 2,815 patients included in the study, 57 (2%) made an unscheduled return visit within 72 hrs. Among the returning patients, 47 (82%) were discharged from the ED, while the remaining ten (18%) were admitted to the hospital, including two ICU admissions and eight ward admissions. The inclusion flow chart is shown in Figure 1.

### 3.2. The Returning versus the Non-Returning Patients and the First Visit versus the Revisit of Returning Patients

The characteristics of the returning and the non-returning patients are presented in Table 1. The mean age of the MHI patients was 33, which was lower than the average age of 39 shown in the administrative data concerning all LCGMH ED patients.

The characteristics of the first visit and the revisit of returning patients are detailed in Table 2. The average length of stay in the ED during the second visit was longer than that for the first visit, albeit with only borderline statistical significance. The triage level and the GCS were about the same. Fourteen of the 57 returning patients received a brain CT, and nine of those 14 patients (64%) were discharged. Twenty-two patients received an X-ray exam during their second visit and then 17 (77%) of them were discharged. Fourteen patients received a blood test during their second visit, and five (36%) of them were discharged.

### 3.3. The Admitted versus the Nonadmitted Returning Patients

Ten of the 57 returning patients were admitted. The characteristics of the “unscheduled return visit with admission” (URVA) patients and the “unscheduled return visit with no admission” (URVNA) patients are presented in Table 3. The URVA patients presented with a higher incidence of vomiting, a GCS of below 15, and a higher triage level and had more blood exams performed during their first visit. Of these ten URVA patients, two received operations for subdural hemorrhage and epidural hemorrhage, respectively. Both of these patients were later discharged uneventfully. Another two patients died due to medical complications resulting from terminal cancers with metastases. A summary of these patients can be seen in Table 4.

## 4. Discussion

In this study, we aimed to identify the factors that are associated with unscheduled return visits and return admissions among MHI patients. This is the first study to investigate the characteristics of this particular population. 82% of the returning MHI patients were discharged again from the ED, which is higher than the overall discharge rate of 57% for returning LCGMH ED patients seen in the administrative data. It would be beneficial in reducing ED overcrowding and minimizing spending on medical resources if some of these return visits were prevented by analyzing the characteristics of returning MHI patients.

In comparing the returning patients with the non-returning patients, we found that the returning patients were older and that they had more comorbidities. These patients also received more blood tests and CT scans during their ED stays. The administrative factors, including the ED overcrowding indicator, different shifts, and physicians' level of experience, did not show significant differences between the two groups. Similar results regarding age being associated with higher rates of return to the ED were shown in previous studies among different patient populations [9, 15–17]. Older patients may have a more atypical presentation, diminished verbal expression, and a complicated past medical history, and it may hence be challenging for physicians to evaluate these patients. Comorbidities also play an important role in evaluating ED trauma patients. Minnee and Wilkinson found that patients over the age of 65 who returned to the ED within three months had an average of 3.4 comorbidities [16]. In a qualitative review of returning patients' medical records, the reasons for their return visit were unrelieved or progressing symptoms, such as vomiting, headache, or altered mental status, as well as anxiety about the delayed diagnosis of serious complications such as intracranial hemorrhage. Discharging such MHI patients with multiple comorbidities only when their symptoms and concerns are completely relieved, providing better discharge instructions, and arranging more timely clinical follow-up appointments may all be considered means of decreasing their return rate.

The distribution of the triage level, vital signs, and symptoms of the first visit and the revisit were identical. For those who returned to the ED, the length of stay of the second visit was longer than that for the first visit, but with only borderline statistical difference. The additional time is possibly spent in providing more exams, reassuring patients and their families, and ensuring more observations [13]. In addition to the length of the ED stay, resources consumption can also be evaluated according to the examinations arranged and the disposition of the second ED visit. Of those who received an image study during their second visit, 64–77% were discharged again from the ED. The additional examinations did not change the disposition of the first visit.

It is both important for the emergency physicians to recognize and treat serious illness and to prevent unnecessary return visits. By analyzing the first ED visit presentations of the URVA and URVNA patients seen in Table 3, we found that the admitted returning patients were more likely to be triaged as level 1 or 2, to have a GCS of <15, to have blood tests performed, and to present with vomiting. Hu et al. showed similar results in their study concerning return visits on the part of the general ED patient population, reporting that old age, high-grade triage, and underlying disease were all associated with a higher rate of URVA [18]. The returning MHI patients who presented with the above characteristics during their first ED visit were more likely to be admitted following a revisit. Emergency physicians should thus be vigilant regarding such returning patients.

The management protocols for traumatic brain injury in Taiwan are based on the Advanced Trauma Life Support (ATLS), developed by the American College of Surgeons (ACS) [19], and the Emergency Trauma Training Course (ETTC), developed by the Taiwan Society of Emergency Medicine (TSEM) [20]. The protocols suggest that if the brain CT is negative and the patient is asymptomatic, awake, alert, and without neurologic deficit, then the patient can be safely discharged [19]. Nevertheless, this guidance makes it difficult to incorporate individual and specific conditions, such as triage level and comorbidities. Triage level is related to vital signs, symptoms, and pain severity [21]. A high triage level usually indicates unstable vital signs, dangerous mechanisms, or severe pain. Comorbidity is associated with a higher incidence of ED revisits, readmission, and mortality [22, 23]. In our study, the two URVA patients who died were both terminal cancer patients. The initial head injury may be a consequence of the underlying cachexic malnourished status, which may later become a deteriorating factor in the terminal status. Two patients suffered from pneumonia or hyponatremia after suffering a MHI, including one ICU admission. This could serve as a reminder that even MHI may cause a diminished daily functional status in vulnerable populations.

## 5. Limitations

There are two limitations to this study. First, patients may return to a different hospital and, as a result, the number of unscheduled return visits may be underestimated. Second, since this is a retrospective electronic data analysis study, it is difficult to standardize the discharge criteria among different doctors. Further protocolized prospective studies may therefore be necessary to minimize individual differences resulting from physicians' practice.

## 6. Conclusions

Minor head injury patients who are of an older age or who exhibit comorbidities are associated with higher rates of unscheduled return ED visits, and so they should only be discharged when their symptoms and concerns are completely relieved, clear discharge instructions have been provided, and a timely clinical follow-up appointment has been arranged. Administrative factors, including ED overcrowding, different shifts, and physicians' level of experience, do not affect the return rate. Those who presented with vomiting, a triage level of 1 or 2, and a GCS score of <15 and who received more blood tests during their first visit were more likely to be admitted when they returned to the ED.

## Figures and Tables

**Table 1:** Characteristics of returning and non-returning minor head injury patients.

	Overall (*N* = 2815)	Returning patient (*N* = 57)	Non-returning patients (*N* = 2758)	*p* value
Male [no. (%)]	1452(52)	30 (53)	1333 (48)	0.52
Age (yr)	33.0 ± 24.3	41.9 ± 24.7	32.8 ± 24.3	0.005^*∗*^
ED factors				
Length of ED stay (hr)	3.1 ± 10.7	3.9 ± 6.5	3.1 ± 10.8	0.35
Physician seniority (yr)	4.3 ± 2.1	4.0 ± 2.3	4.3 ± 2.1	0.38
Total trauma patients on the visit day	76.6 ± 11.9	77.0 ± 12.0	76.6 ± 11.9	0.83
ED visits during nonoffice hours [number (%)]				
Weekend and holiday	1011 (36)	16 (29)	995 (37)	0.21
Night Shifts	1762 (63)	33 (58)	1729 (63)	0.72
Triage, levels 1-2	336 (12)	9 (16)	327 (12)	0.36
Triage level [number (%)]				
Level 1	6 (0.2)	0 (0)	6 (0.2)	
Level 2	330 (12)	9 (16)	321 (12)	
Level 3	1935 (69)	40 (70)	1895 (69)	
Level 4	406 (14)	7 (12)	399 (15)	
Level 5	138 (5)	1 (2)	137 (5)	
Initial vital signs				
Body temperature (°C)	36.5 ± 0.6	36.5 ± 0.6	36.5 ± 0.6	0.82
Respiratory rate (times/min)	20 ± 3	19 ± 2	20 ± 3	0.07
Pulse rate (times/min)	95 ± 23	94 ± 19	95 ± 23	0.78
Mean arterial pressure (mmHg)	107 ± 31	100 ± 37	107 ± 31	0.17
GCS < 15 [number (%)]	101 (4)	3 (5)	98 (4)	0.49
Comorbidity [number (%)]	24 (0.9)	10 (17.5)	14 (0.5)	<0.001^*∗*^
Diabetes mellitus	7 (0.3)	3 (5.3)	4 (0.2)	<0.001^*∗*^
Hypertension	16 (0.6)	9 (15.8)	7 (0.3)	<0.001^*∗*^
Liver cirrhosis	1 (0.04)	0 (0)	1 (0.04)	0.89
End-stage renal disease	4 (0.1)	2 (3.5)	2 (0.1)	<0.001^*∗*^
Examination provided [number (%)]				
Blood test	147 (5)	10 (18)	137 (5)	<0.001^*∗*^
Radiograph	1366 (49)	33 (58)	1333 (48)	0.15
CT	750 (27)	22 (39)	728 (26)	0.04^*∗*^

^*∗*^Statistically significant in comparison between groups; ED, emergency department; GCS, Glasgow Coma Scale; CT, computed tomography.

**Table 2:** Characteristics of the first visit and revisit of returning minor head injury patients.

	First visit	Revisit	*p* value
ED length of stay (hr)	3.9 ± 6.6	8.4 ± 17.3	0.06
ED Physician seniority (yr)	4.0 ± 2.3	4.2 ± 2.8	0.77
Triage level [number (%)]			
Level 1	0 (0)	0 (0)	
Level 2	9 (16)	9 (16)	
Level 3	40 (70)	41 (72)	
Level 4	7 (12)	4 (7)	
Level 5	1 (2)	3 (5)	
Vital Signs			
Body temperature (°C)	36.5 ± 0.6	36.5 ± 0.6	0.96
Pulse rate (times/min)	94 ± 19	86 ± 19	<0.001^*∗*^
Respiratory rate (times/min)	19 ± 2	19 ± 2	0.84
Mean arterial pressure (mmHg)	100 ± 37	92 ± 33	0.005^*∗*^
GCS <15 [number (%)]	3 (5)	3 (5)	1
Symptoms [number (%)]			
Vomiting	12 (21)	19 (34)	0.07
Subcutaneous hematoma	5 (9)	3 (6)	0.16
Examination [number (%)]			
Blood test	10 (18)	14 (25)	
Radiography	33 (58)	22 (39)	
CT	22 (39)	14 (25)	

Comparisons were made using paired *t*-test and McNemar test for continuous and categorical variables, respectively.^*∗*^Statistically significant in comparison between groups. ED, emergency department; GCS, Glasgow Coma Scale; CT, computed tomography.

**Table 3:** First ED visit presentations of admitted and nonadmitted returning minor head injury patients.

	Nonadmission (*N* = 47)	Admission (*N* = 10)	*p* value
Male gender [no. (%)]	21 (45)	6 (60)	0.38
Age (yr)	41.5 ± 25.1	43.8 ± 24.2	0.79
Triage level 1 or 2 in first ED visit [number (%)]	4 (9)	5 (50)	0.005^*∗*^
First visit vital signs			
Body temperature (°C)	36.5 ± 0.6	36.5 ± 0.6	0.72
Heart rate (times/min)	95 ± 19	91 ± 20	0.50
Respiratory rate (times/min)	19 ± 2	20 ± 3	0.42
Mean arterial pressure (mmHg)	101 ± 37	97 ± 37	0.74
GCS<15 [number (%)]	1 (2)	2 (20)	0.02^*∗*^
First visit ED management [number (%)]			
Radiography	28 (60)	5 (50)	0.58
CT	16 (34)	6 (60)	0.13
Blood test	6 (13)	4 (40)	0.04^*∗*^
Suture done	15 (32)	2 (20)	0.24
Consultation	9 (19)	1 (10)	0.32
Mechanism [number (%)]			
MVA Pedestrian	1 (2)	2 (20)	
MVA Car	4 (9)	1 (10)	
MVA Motorcycle	14 (30)	0 (0)	
Fall	21 (45)	6 (60)	
Fight	6 (13)	0 (0)	
Presenting symptoms [number (%)]			
Initial loss of consciousness	11 (23)	5 (50)	0.09
Vomiting	7 (15)	5 (50)	0.01^*∗*^
Subcutaneous hematoma	4 (9)	1 (10)	0.43
Amnesia of any kind	4 (9)	1 (10)	0.43
Send by ambulance [number (%)]	17 (37)	3 (33)	0.84
Comorbidities [number (%)]	11 (23)	3 (30)	0.27
Alcohol use [number (%)]	3 (6)	0 (0)	0.55

^*∗*^Statistically significant in comparison between groups. ED, emergency department; GCS, Glasgow Coma Scale; CT, computed tomography.

**Table 4:** Characteristics of admitted patients in 2nd ED visit.

Number	Age	Gender	Mechanism	Triage	LOS (D)	ILOC	CT	ED diagnosis	Final diagnosis	Operation	ICU/ward	Outcome
1	44	M	MVA	2	7	+	+	(1) Brain concussion (2) C6 right lamina and lateral mass linear fracture	(1) Brain concussion (2) C6 level right lamina fracture	No	Ward	Discharged
2	3	M	Crush	3	5	+	+	Head, chest and abdomen contusion	(1) Brain concussion (2) Liver hematoma	No	Ward	Discharged
3	53	F	Fall	2	25	−	−	Breast cancer with brain mets	(1) Breast ca with multiple mets (2) Pneumonia (3) Brain concussion	No	Ward	Mortality
4	26	M	Fall	3	8	−	−	Headache	(1) Headache, suspected posttraumatic head injury related, need exclude migraine or mood related (2) History of traumatic SDH and SAH (3) Major depression disorder	No	Ward	Discharged
5	44	M	MVA	3	6	+	+	(1) Contusion of face, scalp and neck (2) Scalp laceration (3) Contusion of chest wall	(1) Contusion of chest wall, face, scalp, and neck (2) Multiple subcutaneous emphysema over bilateral buccal, submandibular, neck and upper chest	No	Ward	Discharged
6	82	M	Fall	2	6	−	+	(1) Head injury (2) Lumbar spondylosis	(1) Brain concussion (2) L1-2 osteoporotic compression fracture	No	Ward	Discharged
7	58	F	MVA	2	16	+	−	(1) Head injury (2) Occipital area laceration (3) SDH	(1) Subdural hemorrhage (2) C2 odontoid fx s/p C2 odontoid screws fixation and fusion (3) Neck and bilateral lower limbs contusion	Yes	Ward	Discharged
8	56	F	Fall	3	21	+	+	Brain concussion	(1) Breast ca with multiple mets (2) Hypovolemic shock with multi-organ failure (3) Brain concussion	No	Ward	Mortality
9	62	F	Fall	2	43	−	+	(1) Nasal bone fracture (2) Contusion of face, scalp and neck	(1) Brain concussion with nasal bone fracture (2) Hyponatremia (3) Pneumonia (4) Renal abscess	No	ICU	Discharged
10	11	M	Fall	3	9	−	−	(1) Head injury with local swelling (2) Brain concussion	Left high parietal EDH with mass effect	Yes	ICU	Discharged

LOS (D), length of stay in days; ILOC, initial loss of consciousness; CT, computed tomography; ED, emergency department; MVA, motor vehicle accident; ICU, intensive care unit.
